# Identification and Heterologous Expression of the Albucidin Gene Cluster from the Marine Strain *Streptomyces Albus* Subsp. *Chlorinus* NRRL B-24108

**DOI:** 10.3390/microorganisms8020237

**Published:** 2020-02-10

**Authors:** Maksym Myronovskyi, Birgit Rosenkränzer, Marc Stierhof, Lutz Petzke, Tobias Seiser, Andriy Luzhetskyy

**Affiliations:** 1 Pharmazeutische Biotechnologie, Universität des Saarlandes, 66123 Saarbrücken, Germany; maksym.myronovskyi@uni-saarland.de (M.M.); b.rosenkraenzer@mx.uni-saarland.de (B.R.); s8mcstie@stud.uni-saarland.de (M.S.); 2 BASF SE, 67056 Ludwigshafen, Germany; lutz.petzke@basf.com (L.P.); tobias.seiser@basf.com (T.S.); 3 Helmholtz-Institut für Pharmazeutische Forschung Saarland, 66123 Saarbrücken, Germany

**Keywords:** albucidin, herbicide, nucleoside, biosynthetic gene cluster, heterologous expression, *Streptomyces albus* Del14

## Abstract

Herbicides with new modes of action and safer toxicological and environmental profiles are needed to manage the evolution of weeds that are resistant to commercial herbicides. The unparalleled structural diversity of natural products makes these compounds a promising source for new herbicides. In 2009, a novel nucleoside phytotoxin, albucidin, with broad activity against grass and broadleaf weeds was isolated from a strain of *Streptomyces albus* subsp. *chlorinus* NRRL B-24108. Here, we report the identification and heterologous expression of the previously uncharacterized albucidin gene cluster. Through a series of gene inactivation experiments, a minimal set of albucidin biosynthetic genes was determined. Based on gene annotation and sequence homology, a model for albucidin biosynthesis was suggested. The presented results enable the construction of producer strains for a sustainable supply of albucidin for biological activity studies.

## 1. Introduction

Pesticides play an important role in modern agriculture. Among all chemicals being produced, pesticides are in second place after fertilizers in their extent of use. A total of 2.4 billion kilograms of pesticides were applied worldwide in 2007 [1]. Nevertheless, lack of weed control is still the most topical issue. Among all pests, weeds have the largest negative effect on crop productivity [2,3]. In light of the rapidly increasing evolution of herbicide resistance, the need for new herbicides with new modes of action (MOAs) and safer ecological profiles is growing [4,5].

From all new pesticide active ingredients registered by the Environmental Protection Agency from 1997 to 2010, almost 70% have origins in natural products. Interestingly, only 8% of conventional herbicides are natural product-derived [3,6]. The wide structural diversity of natural products and their small amount of overlap with synthetic compounds imply their potential as lead structures for the development of new pesticides [7,8,9]. This is further confirmed by the phytotoxin literature, which suggests that natural products have many more MOAs than the commercial herbicides currently possess [3].

A novel bleaching herbicide, albucidin, from the strain *Streptomyces albus* subsp. *chlorinus* NRRL B-24108 was discovered in 2009 [10]. In this paper, we present the identification, heterologous expression, and engineering of the albucidin gene cluster. We also propose the biosynthetic route that leads to the production of albucidin. The identified minimal set of biosynthetic genes allows for the straightforward construction of overproducing strains for a high yield albucidin supply for biological activity studies.

## 2. Materials and Methods

### 2.1. General Experimental Procedures

All strains, plasmids and BACs used in this work are listed in Tables S1 and S2. *Escherichia coli* strains were cultured in LB medium [11]. *Streptomyces* strains were grown on soya flour mannitol agar (MS agar) [12] and in liquid tryptic soy broth (TSB; Sigma-Aldrich, St. Louis, MO, USA). For albucidin production, liquid SG medium [13] was used. The antibiotics kanamycin, apramycin, hygromycin, ampicillin and nalidixic acid were supplemented when required.

### 2.2. Isolation and Manipulation of DNA

The previously constructed BAC library of *Streptomyces albus* subsp. *chlorinus* NRRL B-24108 was used [14]. DNA manipulation, *E. coli* transformation and *E. coli*/*Streptomyces* intergeneric conjugation were performed according to standard protocols [11,12,15]. BAC DNA was purified with the BACMAX™ DNA purification kit (Lucigen, Middleton, WI, USA). Restriction endonucleases were used according to the manufacturer’s recommendations (New England Biolabs, Ipswich, MA, USA). All the strains and plasmids are listed in the Tables S1 and S2, respectively.

### 2.3. Metabolite Extraction and Analysis

For metabolite extraction, *Streptomyces* strains were grown in 15 mL of TSB in a 100 mL baffled flask for 1 day, and 1 mL of seed culture was used to inoculate 100 mL of SG production medium in a 500 mL baffled flask. Cultures were grown for 7 days at 28 °C and 180 rpm in an Infors multitron shaker. Albucidin was extracted from the culture supernatant with an equal amount of butanol, evaporated, and dissolved in methanol. Albucidin production was analysed on a Bruker Amazon Speed mass spectrometer coupled to UPLC Thermo Dionex Ultimate 3000 RS. Analytes were separated either on a Waters ACQUITY BEH C18 column (1.7 µm, 2.1 mm × 30 mm) or on a Waters ACQUITY BEH C18 column (1.7 µm, 2.1 mm × 100 mm). Water + 0.1% formic acid and methanol + 0.1% formic acid were used as the mobile phases. For the determination of high-resolution mass, analytes were analysed with a Thermo LTQ Orbitrap XL coupled to UPLC Thermo Dionex Ultimate 3000 RS. Analytes were separated on a Waters ACQUITY BEH C18 column (1.7 µm, 2.1 mm × 100 mm) with water + 0.1% formic acid and methanol + 0.1% formic acid as the mobile phase.

### 2.4. Chemical Mutagenesis

One millilitre of spore suspension of *Streptomyces albus* subsp. *chlorinus* NRRL B-24108 was inoculated into 100 mL of SG medium in a 500 mL baffled flask and cultivated overnight at 28 °C and 180 rpm. The pH of the culture was adjusted to 8.5 with 1 M NaOH. Ten millilitres of culture was transferred into three 50 mL falcon tubes. Then, 64 mg of wet NTG was dissolved in 16 mL of water. Next, 6.666 mL, 4.285 mL and 1.765 mL of NTG stock solution were added to the tubes containing culture to reach final NTG concentrations of 800 µg/mL, 600 µg/mL and 300 µg/mL, respectively. The samples were incubated at 28 °C for 30 min in the overhead shaker. The mycelium was precipitated by centrifugation, and the supernatant was discarded. The mycelium was washed twice with 5% sodium thiosulfate solution. The treated samples were plated on MS agar plates and cultivated for 14 days at 28 °C. The spores were washed with water and plated in dilutions on MS agar. The plates with spore dilutions were incubated for 10 days at 28 °C. Single colonies were picked on 30 mm plates with SG agar. The plates were incubated for 14 days at 28 °C. Agar blocks were cut out from the plates and transferred into 2 mL tubes. Albucidin was extracted from the agar blocks with 500 µL of butanol for 48 h. The extracts were analysed using HPLC-MS.

### 2.5. Albucidin Isolation and ^1^H-NMR Spectroscopy

*Streptomyces albus* 1K1 was grown in 10 L of SG medium, and albucidin was extracted with butanol. The dry extract was dissolved in 50 mL of water containing 5% acetonitrile and 0.1% formic acid. The extract was loaded onto 3 C18 SPE columns (Discovery DSC-18 SPE 52607-U) equilibrated with 5% acetonitrile in water with 0.1% formic acid. The flowthrough was collected. The columns were washed twice with 12 mL of 5% acetonitrile containing 0.1% formic acid. The flowthrough and the wash fractions were combined and evaporated in a rotary evaporator. The presence of albucidin was detected by HPLC-MS.

The dry material after the SPE purification step was dissolved in methanol and used for size-exclusion chromatography. Separation was performed on a glass column (30 mm × 1000 mm) packed with Sephadex LH-20 and methanol as the mobile phase. Fractions containing albucidin were identified by HPLC-MS. Albucidin-containing fractions were combined and evaporated. The dry extract was dissolved in 5 mL of 5% methanol in water containing 10 mM potassium phosphate buffer pH 6.4.

HPLC separation was performed on a preparative HPLC Thermo Dionex Ultimate 3000 equipped with a Macherey Nagel Nucleodur HTec C18 column (5 µm, 21 mm × 150 mm). A 10 mM potassium phosphate buffer (pH 6.4) was used as solvent A, and 50% methanol in 10 mM phosphate buffer (pH 6,4) was used as solvent B. The following gradient at a flowrate of 15 mL/min was used for separation: 0 min–13% B, 20 min—25% B, 21 min–25% B, 24 min–100% B, 25 min–100% B, 28 min–13% B, 29 min–13% B. Albucidin eluted at 18 min. The albucidin-containing fractions were pooled and evaporated.

For the final purification step, the dry material after HPLC purification was dissolved in 5 mL of water and loaded onto a Sephadex LH-20 column (30 mm × 450 mm) previously equilibrated with water. Water was used as the mobile phase. The fractions containing albucidin were identified by HPLC-MS, pooled and evaporated.

The ^1^H-NMR spectra were recorded on a Bruker Avance 500 spectrometer (Bruker, BioSpin GmbH, Rheinstetten, Germany) at 300 K equipped with a 5 mm BBO probe using deuterated trifluoroacetic acid (Deutero, Kastellaun, Germany) as the solvent containing tetramethylsilane (TMS) as a reference. Albucidin was measured in deuterated water (Deutero, Kastellaun, Germany). The chemical shifts are reported in parts per million (ppm) relative to TMS. All spectra were recorded with the standard ^1^H pulse program using 128 scans. The structure of albucidin was confirmed by comparison of the recorded ^1^H NMR data (Figure S1) with published data [10].

### 2.6. Construction of the 1K1 BAC Derivatives

The derivatives of 1K1 BAC with gene deletions were constructed using the RedET approach. For this, the antibiotic resistance marker was amplified by PCR with primers harbouring overhang regions complementary to the boundaries of the DNA to be deleted. The amplified fragment was used for recombineering of the BAC. The recombinant BACs were analysed by restriction mapping and sequencing. The primers used for recombineering purposes are listed in Table S3.

For the construction of BAC 1K1_LS, the ampicillin marker from pUC19 was amplified with the primers LS-F/LS-R. For the construction of the BAC 1K1_RS, the hygromycin marker from pACS-hyg [16] was amplified with the primers RS-F/RS-R. For the construction of the BACs 1K1_KO14, 1K1_KO15 and 1K1_KO16, the ampicillin cassette was amplified with the pairs of primers KO14-F/KO14-R, KO15-F/KO15-R and KO16-F/KO16-R, respectively. For the construction of the BACs 1K1_KO7, 1K1_KO8, 1K1_KO9, 1K1_KO10, 1K1_KO11, 1K1_KO12 and 1K1_KO13, the ampicillin cassette was amplified with the pairs of primers KO7-F/KO7-R, KO8-F/KO8-R, KO9-F/KO9-R, KO10-F/KO10-R, KO11-F/KO11-R, KO12-F/KO12-R and KO13-F/KO13-R, respectively.

BAC 1K1_alb_act was constructed in two steps. First, 1K1_RS2 BAC was constructed from 1K1 using an ampicillin marker amplified with primers RS2-F/RS2-R. Then BAC 1K1_alb_act was constructed by recombineering the BAC 1K1_RS2 using a hygromycin marker from pACS-hyg amplified with the primers ACT-F/ACT-R.

### 2.7. Genome Mining and Bioinformatics Analysis

The *S. albus* subsp. *chlorinus* genome was screened for secondary metabolite biosynthetic gene clusters using the antiSMASH online tool [17] and the software Geneious [18]. The genomic sequence of the albucidin producer *S. albus* subsp. *chlorinus* NRRL B-24108 was deposited in GenBank under accession number VJOK00000000 [14].

## 3. Results and Discussion

### 3.1. Identification of the Albucidin Biosynthetic Gene Cluster

The aim of this study was to identify the biosynthetic genes leading to the production of the nucleoside phytotoxin albucidin. For this purpose, the genome sequence of the producer strain of *Streptomyces albus* subsp. *chlorinus* NRRL B-24108 was analysed by genome-mining software [17]. This analysis led to the identification of several putative nucleoside clusters. To prove the involvement of these candidate clusters in albucidin production, they were heterologously expressed in a genetically engineered cluster-free strain *Streptomyces albus* Del14 [19] and in *Streptomyces lividans* TK24 [20]. No albucidin production was detected in the extracts of the obtained strains, indicating that either the expressed clusters were not involved in the biosynthesis of albucidin or they were not expressed in the heterologous host environment. The inactivation of the candidate clusters in the natural albucidin producer was not feasible because the strain is refractory to genetic manipulation. Considering the difficulties in identifying the albucidin gene cluster using conventional methods, an alternative approach using chemical mutagenesis was chosen.

**Figure 1:**
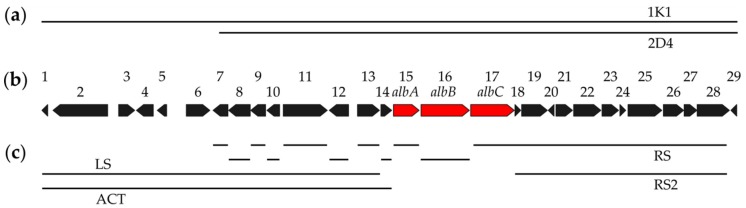
Chromosomal fragment of *S. albus* subsp. *chlorinus* NRRL B-24108 with the albucidin biosynthetic genes. (**a**) Schematic representations of DNA fragments cloned in BACs 1K1 and 2D4; (**b**) The genes encoded within the fragment cloned in BAC 1K1. The *albA–C* operon is marked in red; and (**c**) Overview of the performed deletions within BAC 1K1.

For chemical mutagenesis of the albucidin-producing strain *S. albus* subsp. *chlorinus* NRRL B-24108, 1-methyl-3-nitro-1-nitrosoguanidine (NTG) was used. The strain in the exponential growth stage was treated with various NTG concentrations (800 µg/mL, 600 µg/mL and 300 µg/mL) for 30 min. After mutagenesis, the cells were washed with 5% thiosulfate solution and plated in dilutions on MS-agar medium for segregation of mutations. The spores of the obtained mutant populations were washed and plated on MS-agar plates in dilutions to obtain single colonies. Altogether, 4000 individual mutants were analysed for albucidin production. The mutants were cultivated on individual plates with the production medium SG agar. The metabolites were extracted with butanol, and albucidin production was assayed by HPLC-MS. Eight mutants that lost the ability to produce albucidin were identified in the course of this screening: 6-238, 6-260, 6-389, 6-444, 6-612, 6-892, 8-610 and 8-639. The genomic DNA of the obtained zero mutants was sequenced using Illumina technology. The point mutations in the genomes of the mutants were detected by mapping the sequencing reads to the reference genome of the wild type albucidin producer. Up to 100 transition mutations were identified in the genomes of the mutant strains. By comparing the mutation patterns of the separate mutants, a short genomic region was identified that was affected by point mutations in all analysed zero mutants, implying its potential involvement in albucidin production (Figure S2). The identified region contains two genes, *SACHL2_05525* and *SACHL2_05524*, which encode putative radical SAM proteins and were named *albA* and *albB* (Table 1, Figure 1b). The genes constitute a putative operon with the third gene *SACHL2_05523*, which was named *albC*. The *albC* gene encodes a putative ribonucleoside-triphosphate reductase and was not affected by point mutations in the analysed zero mutants of *S. albus* subsp. *chlorinus* NRRL B-24108. The identified genes *albA* and *albB* were not a part of the nucleoside gene clusters previously identified by genome mining and analysed in this study. Interestingly, these genes were located within the DNA fragment annotated by genome mining software as a putative NRPS gene cluster.

To determine whether the identified genes *albA* and *albB* encode albucidin biosynthetic enzymes, a BAC 1K1 containing the abovementioned genes (Figure 1a) was selected from the genomic library of *S. albus* subsp. *chlorinus* NRRL B-24108. BAC 1K1 was transferred into the heterologous host strains *S. albus* Del14 and *S. lividans* TK24 by conjugation, and the production profile of the obtained strains *S. albus* 1K1 and *S. lividans* 1K1 was analysed by HPLC-MS. The production of the compound with a high-resolution mass corresponding to albucidin could be detected in the extracts of *S. albus* 1K1 (Figure S3). No production could be detected in *S. lividans* 1K1.

**Figure 2:**
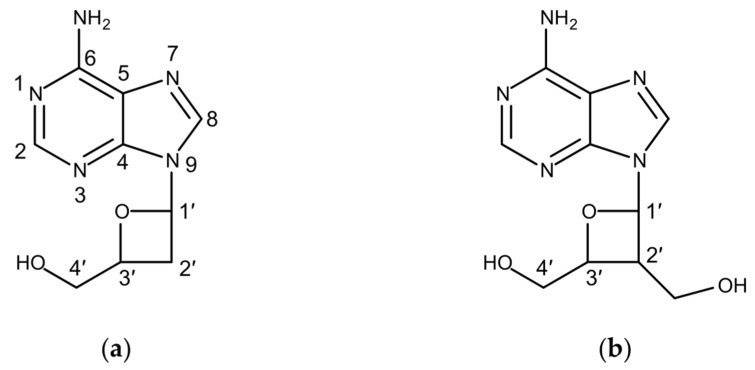
The structures of (**a**) albucidin and (**b**) oxetanocin A.

Due to the lack of an albucidin standard, we set out to purify the compound identified in the extracts of *S. albus* 1K1 for structure elucidation studies by NMR spectroscopy. The *S. albus* 1K1 strain was cultivated in 10 L of SG medium for 7 days. The culture supernatant was extracted with equal amount of butanol, and the obtained extract was concentrated under vacuum. Four milligrams of the compound was purified using size exclusion and reverse phase chromatography and used for subsequent NMR studies. Analysis of the recorded NMR spectra of the purified compound unequivocally demonstrated its identity as albucidin (Figure S1 and Figure 2a).

The production of albucidin by *S. albus* 1K1 gives evidence that the genes *albA* and *albB* identified by chemical mutagenesis encode albucidin biosynthetic genes. The lack of albucidin production by *S. lividans* 1K1 can be explained by differences in regulatory networks of the *S. albus* Del14 and *S. lividans* TK24 strains.

### 3.2. Identification of the Minimal Set of Albucidin Biosynthetic Genes

BAC 1K1, which leads to the production of albucidin under expression in the heterologous host *S. albus* Del14, contains a 32 kb chromosomal fragment from the natural albucidin producer *S. albus* subsp. *chlorinus* NRRL B-24108. Twenty-nine open reading frames were annotated in this 32 kb region (Table 1, Figure 1b). Of these genes, only two, *albA* and *albB*, were affected by point mutations in albucidin zero mutants identified in the course of the chemical mutagenesis studies. These two genes constitute a putative operon with the gene *albC,* implying that either only the genes *albA* and *albB* are necessary for albucidin production or that all three gene within the operon are required. To experimentally determine the minimal set of albucidin biosynthetic genes, a series of gene deletions was performed within the cloned region of 1K1 BAC.

The genes *albA–C* are located in the middle part of the chromosomal fragment cloned in 1K1 BAC. The *alb* operon is preceded by the genes *SACHL2_05539–SACHL2_05526*, followed by the genes *SACHL2_05522–SACHL2_05511* (Table 1). For the sake of simplicity, the 29 genes *SACHL2_05539–SACHL2_05511* cloned in the BAC 1K1 will be designated in the text according to their sequence number (1 to 29). (Figure 1b) To determine which genes within the 1K1 cloned fragment are essential for albucidin production, five deletions (LS, KO14, KO15, KO16 and RS) were performed in the 1K1 BAC yielding the BACs 1K1_LS, 1K1_KO14, 1K1_KO15, 1K1_KO16 and 1K1_RS (Figure 1c). In BAC 1K1_LS, the left shoulder encompassing genes 1–13 was substituted by an ampicillin resistance marker (Figure 1c). The genes 14, *albA* (gene 15) and *albB* (gene 16) were substituted with the ampicillin resistance gene in BACs 1K1_KO14, 1K1_KO15 and 1K1_KO16, respectively (Figure 1c). In the BAC 1K1_RS, the right shoulder encompassing genes *albC* (gene 17)-28 was substituted by the hygromycin resistance marker (Figure 1c). The constructed BACs were transferred separately into the *S. albus* Del14 strain by conjugation, and the albucidin production of the resulting strains was assayed by HPLC-MS.

The deletion of gene 14 did not affect albucidin production in *S. albus* 1K1_KO14 (Figure S4B). As expected from the results of chemical mutagenesis, inactivation of the genes *albA* (gene 15) and *albB* (gene 16) completely abolished albucidin production in the strains *S. albus* 1K1_KO15 and *S. albus* 1K1_KO16 (Figure S4C,D). This unambiguously demonstrates the essential role of the genes *albA* and *albB* in albucidin biosynthesis.

Deletion of the genes *albC* (gene 17)-28 did not affect albucidin production by the *S. albus* RS strain (Figure S4F). It was expected from the gene annotation and results of chemical mutagenesis that the genes 18–28 do not participate in albucidin biosynthesis. However, the dispensability of the gene *albC* (gene 17) is surprising since it belongs to the same operon as the essential genes *albA* (gene 15) and *albB* (gene 16). The deletion of the *albC* gene (gene 17) might be cross-complemented by an unidentified gene in the genome of the host strain *S. albus* Del14.

Albucidin production was heavily abolished in the strain *S. albus* 1K1_LS (Figure S4E), implying that at least one of the genes 1–13 that were deleted in the BAC 1K1_LS might be essential for albucidin biosynthesis. No genes encoding regulatory proteins or structural enzymes that might participate in nucleoside biosynthesis were identified in close proximity to the *albA–C* operon. To identify the genes within the deleted LS region that influence albucidin production, a BAC 2D4 was isolated from the genomic library of *S. albus* subsp. *chlorinus* NRRL B-24108. The chromosomal fragment cloned in BAC 2D4 overlaps with the fragment cloned in BAC 1K1 and covers the *albA–C* operon (Figure 1a). In contrast to 1K1, 2D4 BAC lacks genes 1–6, which are present in the deleted LS region. BAC 2D4 was transferred into *S. albus* Del14. Albucidin production could be detected in the extracts of the obtained strain *S. albus* 2D4 by HPLC-MS (Figure S5). This indicates that the genes 1–6 within the LS region are not involved in albucidin production and that one of the genes among 7–13 is responsible for the abolishment of albucidin production in *S. albus* 1K1_LS.

To identify which of the genes 7–13 is involved in albucidin biosynthesis, each of them was individually substituted by an ampicillin resistance marker in 1K1 BAC yielding 1K1_KO7, 1K1_KO8, 1K1_KO9, 1K1_KO10, 1K1_KO11, 1K1_KO12 and 1K1_KO13 (Figure 1b,c). The constructed BACs were transferred into the *S. albus* Del14 strain, and the albucidin production was analysed. The albucidin production levels of all obtained strains, except *S. albus* 1K1_KO12, were in the range of *S. albus* 1K1 harbouring the unmodified BAC (Figure S6). Albucidin production was abolished in *S. albus* 1K1_KO12 (Figure S6G), indicating that gene 12 is responsible for the detrimental effect of the LS deletion on albucidin biosynthesis. No enzymatic activity could be assigned to the peptide product of gene 12 using blast analysis. The product also did not show homology to any known regulatory protein. Considering this, it was proposed that only the genes *albA* and *albB* encode structural enzymes essential for albucidin production in the heterologous host *S. albus* Del14 and that the product of the gene 12 elicits a regulatory effect on transcription of the *albA–C* operon through a mechanism that is not understood. To prove this, a BAC 1K1_alb_act was constructed containing only *albA–C* genes under the control of a strong promoter. The genes downstream of the *albA–C* operon (genes 18–28) were substituted with the ampicillin resistance gene and the genes upstream of the operon (genes 1–14) were substituted with the hygromycin resistance gene (Figure 1c). The hygromycin resistance gene used was under the control of the strong synthetic promoter TS81 and did not contain a terminator at its 3’-end [21]. The insertion of the hygromycin marker in front of the *albA–C* genes was performed in the orientation, which enabled their read-through from the TS81 promoter and their transcriptional activation. The constructed BAC 1K1_alb_act was transferred into the heterologous host strain *S. albus* Del14. The production of albucidin was detected in the extracts of the obtained strain *S. albus* 1K1_alb_act by HPLC-MS (Figure S7). Three times increase of albucidin production was observed in the strain *S. albus* 1K1_alb_act compared to *S. albus* 1K1 containing non-modified albucidin cluster (Figure S7). Taking into account that the total recovered albucidin yield from the *S. albus* 1K1 strain was approximately 0.4 mg/L, the calculated albucidin production by *S. albus* 1K1_alb_act corresponded to 1.2 mg/L. The albucidin production rate of 2 mg/L was reported for the original producer *Streptomyces albus* subsp. *chlorinus* NRRL B-24108 [10].

Albucidin production by the strain *S. albus* 1K1_alb_act clearly demonstrates that the genes *albA* and *albB* constitute the minimal set of the genes required for albucidin biosynthesis in heterologous host *S. albus* Del14. The role of the gene *albC* in albucidin biosynthesis is not completely understood. Because *albC* constitutes a single operon with *albA* and *albB* and its product shows homology to nucleotide biosynthetic enzymes, it cannot be completely excluded that the *albC* is involved in albucidin production in the natural producer. However, the deletion of *albC* has no effect on albucidin production in heterologous host.

The identification of the minimal set of albucidin biosynthetic genes allows its expression in various heterologous chassis strains as well as rational construction of albucidin overproducers. The engineering of the albucidin biosynthetic genes can be performed in *E. coli* and the obtained constructs can be heterologously expressed in *Streptomyces* hosts. In contrast to the genetically intractable original albucidin producer *Streptomyces albus* subsp. *chlorinus* NRRL B-24108, commonly used heterologous strains possess a well-established toolkit for their genetic manipulation. This opens the possibility to engineer their metabolic network to increase the intracellular levels of biosynthetic precursors and therefore to increase the production yields. The heterologous strains are often characterized by the simplified metabolic background which provides better detection limits for heterologously expressed compounds than the original producers, higher product yields and simplified downstream processing. Construction of the albucidin overproducers based on heterologous expression hosts is not necessarily limited to a rational approach. The chassis strains expressing heterologous cluster may be also subjected to classical mutagenesis and screening for overproducing clones.

### 3.3. Proposed Biosynthetic Pathway of Albucidin

Structurally, albucidin is closely related to oxetanocin A (Figure 2b), which has been isolated from the culture of *Bacillus megaterium* NK84-0218 [22]. Both compounds are the only known naturally occurring nucleosides featuring four membered oxetane rings in their structure. From a structural view, albucidin is 2′-dehydroxymethyl oxetanocin A. Two genes, *oxsA* and *oxsB,* encoding a putative HD domain phosphohydrolase and a cobalamin-dependent S-adenosylmethionine radical enzyme have been reported to be responsible for oxetanocin biosynthesis [23]. dAMP, dADP and dATP were identified as direct oxetanocin precursors [24]. The product of *oxsB* catalyses the contraction of the deoxyribose ring, while the product of *oxsA* is responsible for the removal of one or multiple phosphates from a phosphorylated 2′-deoxyadenosine derivative [24,25]. Through the simultaneous actions of OxsA and OxsB, the phosphorylated 2′-deoxyadenosine is converted to the oxetanocin A precursor, its aldehyde form, which must be reduced to complete biosynthesis [24]. This reaction is not encoded by the genes within the oxetanocin A cluster and is likely to be carried by an unidentified enzyme of *B. megaterium* NK84-0218.

**Figure 3:**
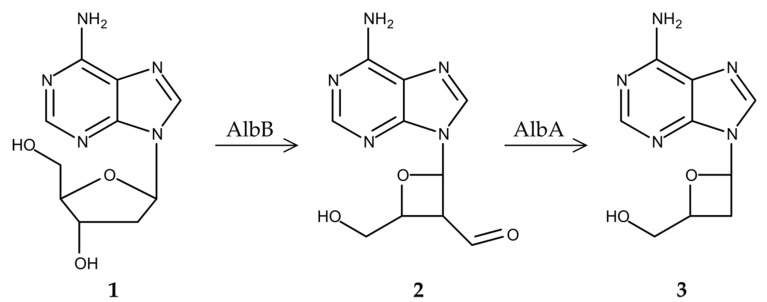
The proposed scheme of albucidin biosynthesis. 2′-Deoxyadenosine (**1**) is converted into the aldehyde form of oxetanocin A (**2**) by the product of *albB*. The latter is then converted into albucidin (**3**) by the product of *albA*.

Gene inactivation studies have given evidence that two genes, *albA* and *albB*, are required for the production of albucidin. Both genes encode putative SAM radical proteins. At the protein level, the *albA* gene shows homology to biotin synthases and the *albB* gene shows homology to the product of the oxetanocin biosynthetic gene *oxsB*. Despite the high structural similarity of albucidin and oxetanocin A, the homologue of the second oxetanocin biosynthetic gene *oxsA* cannot be found within the albucidin cluster or in the genome of albucidin producer *S. albus* subsp. *chlorinus* NRRL B-24108. The homology of the *albB* gene to *oxsB* implies that the product of *albB* might also be responsible for the ring contraction reaction in albucidin biosynthesis. However, the structural differences between albucidin and oxetanocin and the absence of an *oxsA* homologue imply substantial differences in biosynthetic routes leading to the biosynthesis of the nucleosides. Due to the lack of an *oxsA* homologue that is responsible for the dephosphorylation of adenine deoxyribonucleotides during oxetanocin biosynthesis, we propose that deoxyadenosine is used instead of dAMP, dADP or dATP as a precursor for albucidin production. The product of *albB* is likely responsible for the contraction of the deoxyribose ring of deoxyadenosine (Figure 3) in a similar manner as its homologue OxsB catalyses oxetane ring formation in oxetanocin A biosynthesis [24]. As a result of this reaction, the aldehyde form of oxetanocine A is formed. The conversion of the latter into albucidin is likely to be catalysed by the product of *albA,* which removes the aldehyde group from the 2’-position (Figure 3).

In this paper, we report the identification, cloning, and heterologous expression of the albucidin biosynthetic gene cluster from *Streptomyces albus* subsp. *chlorinus* NRRL B-24108. Albucidin is a nucleoside phytotoxin featuring a rare oxetane ring in its structure. This metabolite shows herbicidal activity against a broad spectrum of grass and broadleaf weeds. In treated plants, albucidin induces metabolic perturbation, chlorosis, and bleaching [10]. The exact MOA of the compound remains unknown. The identification of the albucidin cluster presented in this paper enables biosynthetic studies of albucidin, optimization of its production as well as albucidin supply for the determination of its MOA.

## 4. Patents

(WO2018224939) Gene cluster for the biosynthesis of albucidin.

## Figures and Tables

**Table 1:** Genes encoded within the chromosomal fragment cloned in BAC 1K1.

Gene	Locus Tag ^1^	Putative Function
1	*SACHL2_05539*	Hypothetical protein
2	*SACHL2_05538*	ABC transporter
3	*SACHL2_05537*	Transcriptional regulatory protein LiaR
4	*SACHL2_05536*	Hypothetical protein
5	*SACHL2_05535*	Hypothetical protein
6	*SACHL2_05534*	beta-lactamase/D-alanine carboxypeptidase
7	*SACHL2_05533*	Chondramide synthase
8	*SACHL2_05532*	Hypothetical protein
9	*SACHL2_05531*	Hypothetical protein
10	*SACHL2_05530*	Thymidylate kinase
11	*SACHL2_05529*	Pyruvate, phosphate dikinase
12	*SACHL2_05528*	Hypothetical protein
13	*SACHL2_05527*	Hypothetical protein
14	*SACHL2_05526*	Hypothetical protein
15; *albA*	*SACHL2_05525*	Biotin synthase, radical SAM protein
16; *albB*	*SACHL2_05524*	Radical SAM protein
17; *albC*	*SACHL2_05523*	Ribonucleoside-triphosphate reductase
18	*SACHL2_05522*	Tyrocidine synthase 3
19	*SACHL2_05521*	Plipastatin synthase, subunit A
20	*SACHL2_05520*	Acyl carrier protein
21	*SACHL2_05519*	Demethylmenaquinone methyltransferase
22	*SACHL2_05518*	Linear gramicidin synthase, subunit D
23	*SACHL2_05517*	Hypothetical protein
24	*SACHL2_05516*	Acyl carrier protein
25	*SACHL2_05515*	Fatty-acid--CoA ligase
26	*SACHL2_05514*	Ribonucleotide-diphosphate reductase
27	*SACHL2_05513*	Hypothetical protein
28	*SACHL2_05512*	Tyrocidine synthase 3
29	*SACHL2_05511*	Hypothetical protein

^1^ The locus tags refer to the genome sequence of *S. albus* subsp. *chlorinus* NRRL B-24108 available under GenBank accession number VJOK00000000.

## Supplementary Materials

The following are available online at https://www.mdpi.com/2076-2607/8/2/237/s1, Figure S1: ^1^H NMR spectrum of albucidin (500 MHz), Figure S2: The genes *albA* and *albB* with the mapped point mutations identified in the course of NTG-mutagenesis, Figure S3: High resolution HPLC-MS analysis of albucidin production by *Streptomyces albus* 1K1, Figure S4: HPLC-MS analysis of albucidin production by *Streptomyces albus* strain harboring 1K1 BAC and its derivatives with gene deletions, Figure S5: HPLC-MS analysis of albucidin production by *Streptomyces albus* 1K1 BAC (**a**) and *Streptomyces albus* 2D4 BAC (**b**), Figure S6: HPLC-MS analysis of albucidin production by *Streptomyces albus* strain harboring 1K1 BAC and its derivatives with gene deletions, Figure S7: HPLC-MS analysis of albucidin production by *Streptomyces albus* strain harboring full length 1K1 BAC (**a**) and minimized 1K1_alb_act BAC, containing only transcriptionally activated *albA–C* operon (**b**), Table S1: Bacterial strains used in the study, Table S2: Plasmids and BACs used in the study, Table S3: Primers used in this study. References [26,27] are cite d in the supplementary materials.
