# Supplementary material for: Identification and Heterologous Expression of the Albucidin Gene Cluster from the Marine Strain Streptomyces Albus Subsp. Chlorinus NRRL B-24108
Source: Microorganisms. 2020 Feb 10;8(2):237. doi: 10.3390/microorganisms8020237 (PMC7074753; doi:10.3390/microorganisms8020237)
Supplement: Supplementary file 1 [file microorganisms-08-00237-s001.pdf]

microorganisms

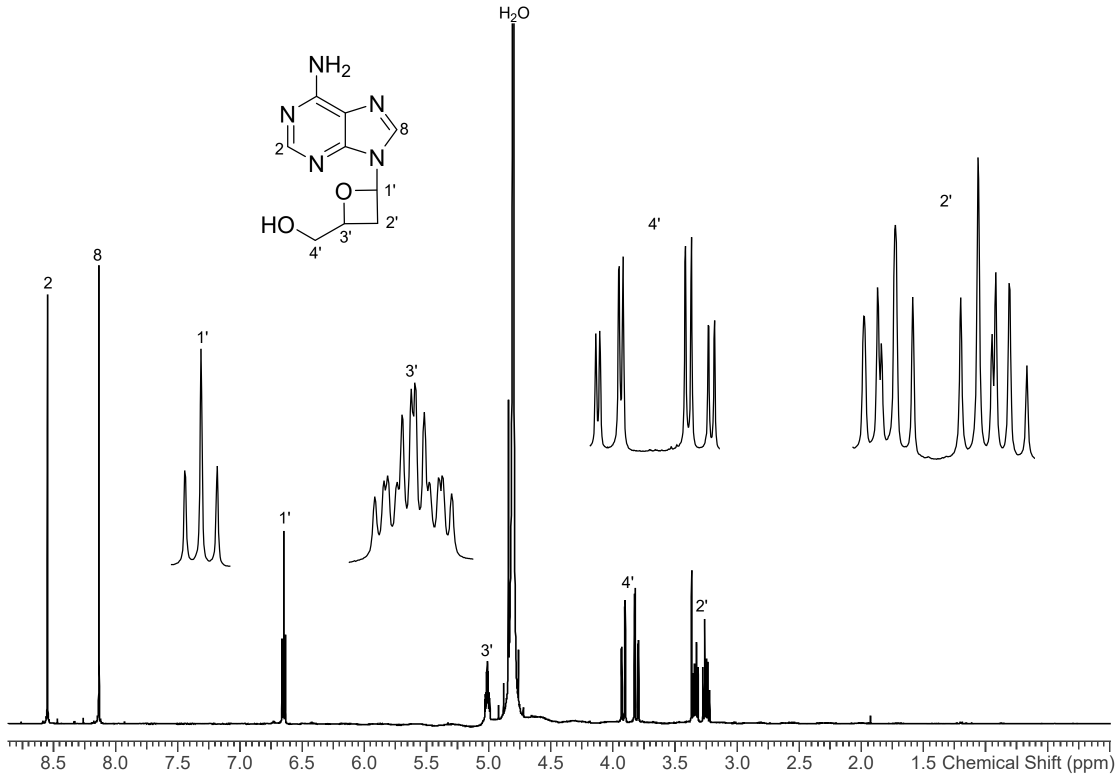

Figure S1. ^1H NMR spectrum of albucidin (500 MHz). ^1H NMR (D_2O) δ 8.55 (s, 1H), 8.13 (s, 1H), 6.65 (t, *J* = 6.9, 1H), 5.01 (m, 1H), 3.92 (dd, *J* = 2.3, 13.5, 1H), 3.81 (dd, *J* = 3.5, 13.5, 1H), 3.33 (m, 1H), 3.25 (m, 1H).

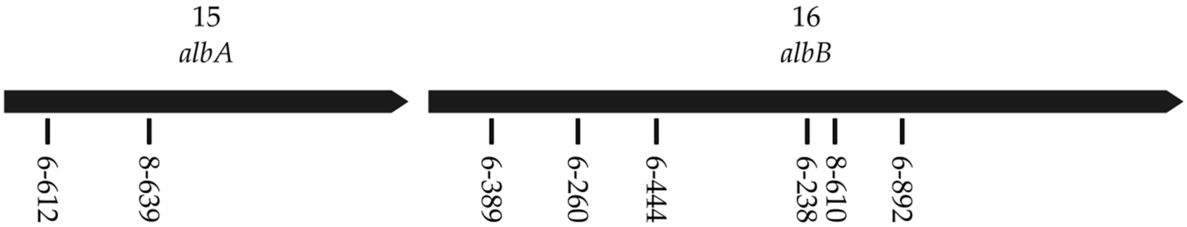

Figure S2. The genes *albA* and *albB* with the mapped point mutations identified in the course of NTG-mutagenesis. The positions of the mutations are shown as vertical lines. The mutant 6-612 contains a transition mutation G→A in the position 133 of *albA*. The mutant 8-639 contains a transition mutation G→A in the position 431 of *albA*. The mutant 6-389 contains a transition mutation G→A in the position 190 of *albB*. The mutant 6-260 contains a transition mutation G→A in the position 445 of *albB*. The mutant 6-444 contains a transition mutation G→A in the position 677 of *albB*. The mutant 6-238 contains a transition mutation G→A in the position 1118 of *albB*. The mutant 8-610 contains a transition mutation C→T in the position 1205 of *albB*. The mutant 6-892 contains a transition mutation G→A in the position 1403 of *albB*.

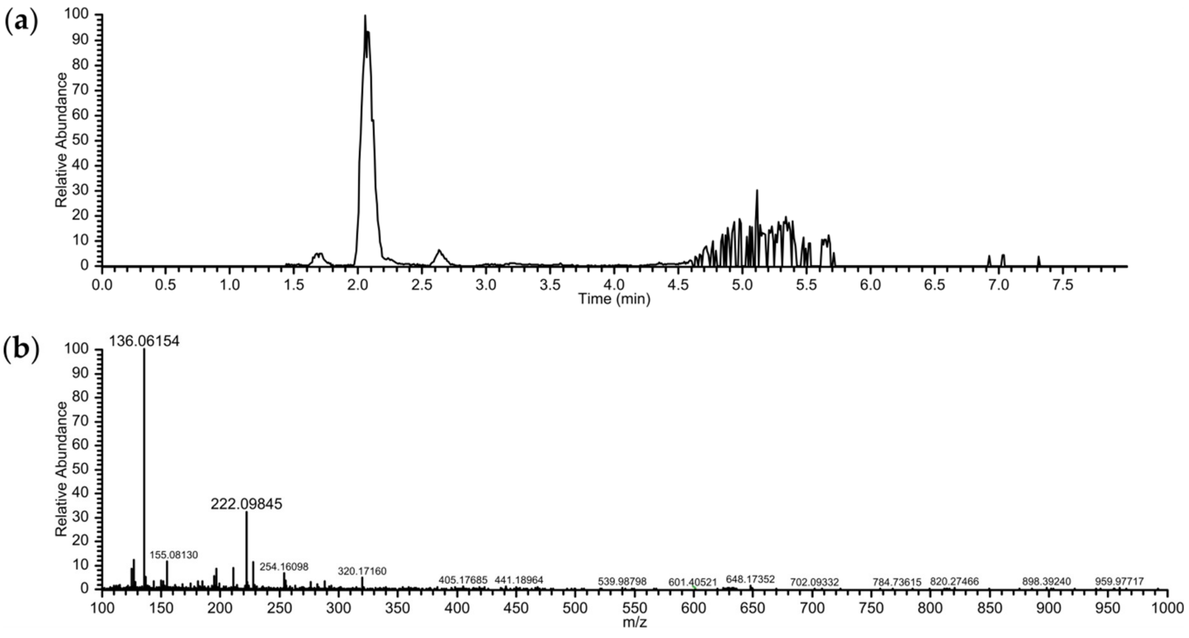

Figure S3. High resolution HPLC-MS analysis of albucidin production by *Streptomyces albus* 1K1. (**a**) Extracted ion chromatogram (222.00 ± 0.15 Da) of crude extract from *S. albus* 1K1. (**b**) High resolution mass spectrum of the peak corresponding to albucidin at t_R = 2.1 min.

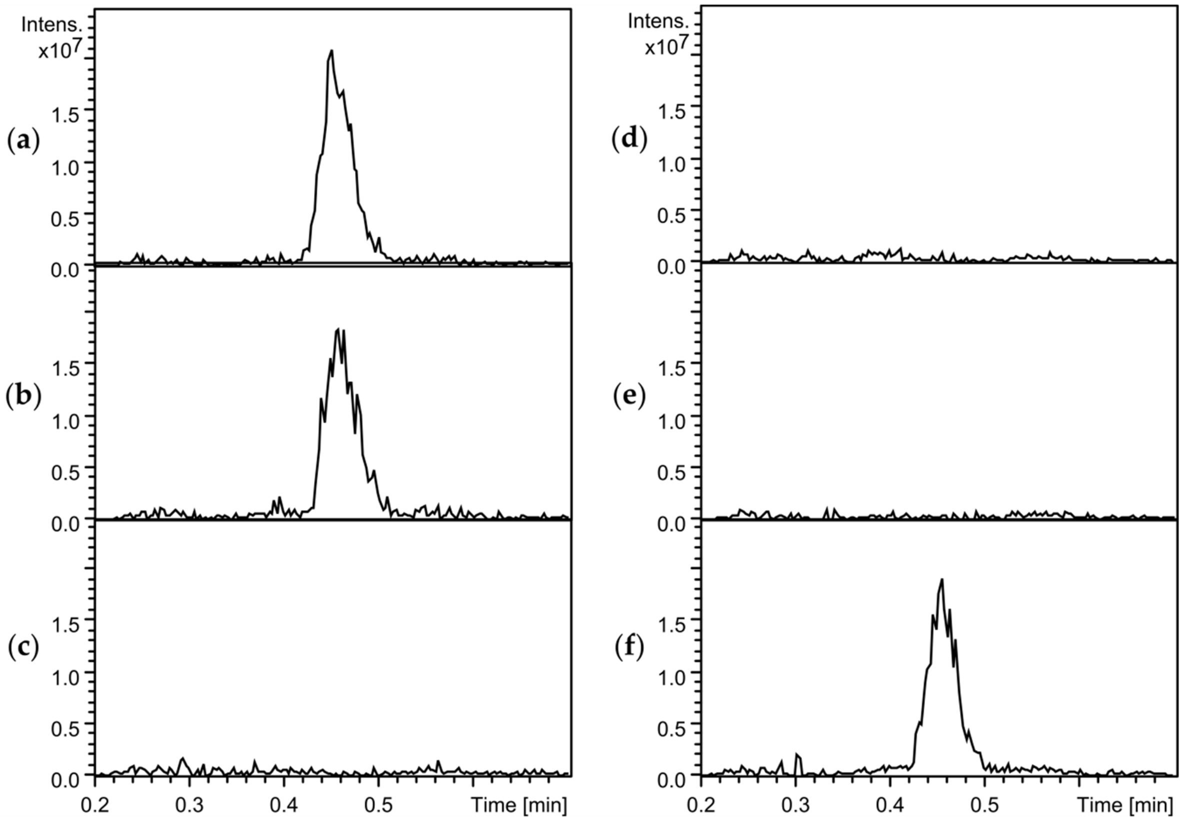

Figure S4. HPLC-MS analysis of albucidin production by *Streptomyces albus* strain harboring 1K1 BAC and its derivatives with gene deletions. Extracted chromatograms (222.00 ± 0. 5 Da) of crude extracts from *S. albus* 1K1 (**a**), *S. albus* 1K1_KO14 (**b**), *S. albus* 1K1_KO15 (**c**), *S. albus* 1K1_KO16 (**d**), *S. albus* 1K1_LS (**e**), *S. albus* 1K1_RS (**f**) are shown.

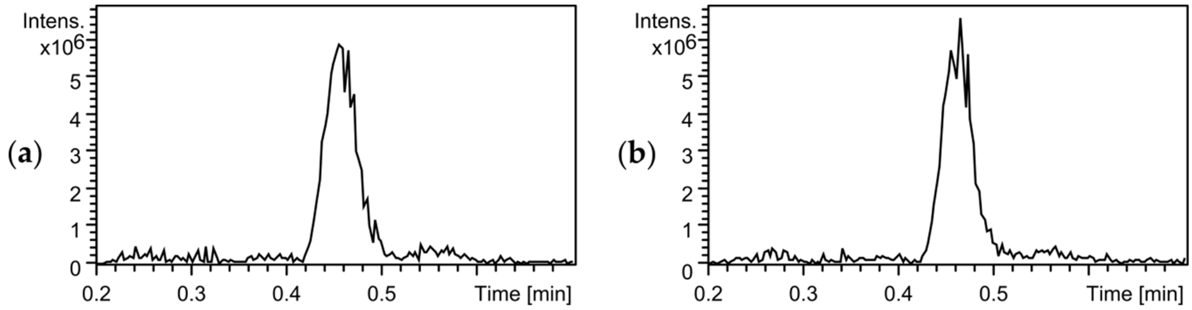

Figure S5. HPLC-MS analysis of albucidin production by *Streptomyces albus* 1K1 BAC (**a**) and *Streptomyces albus* 2D4 BAC (**b**). Extracted chromatograms (222.00 ± 0. 5 Da) of crude extracts are shown.

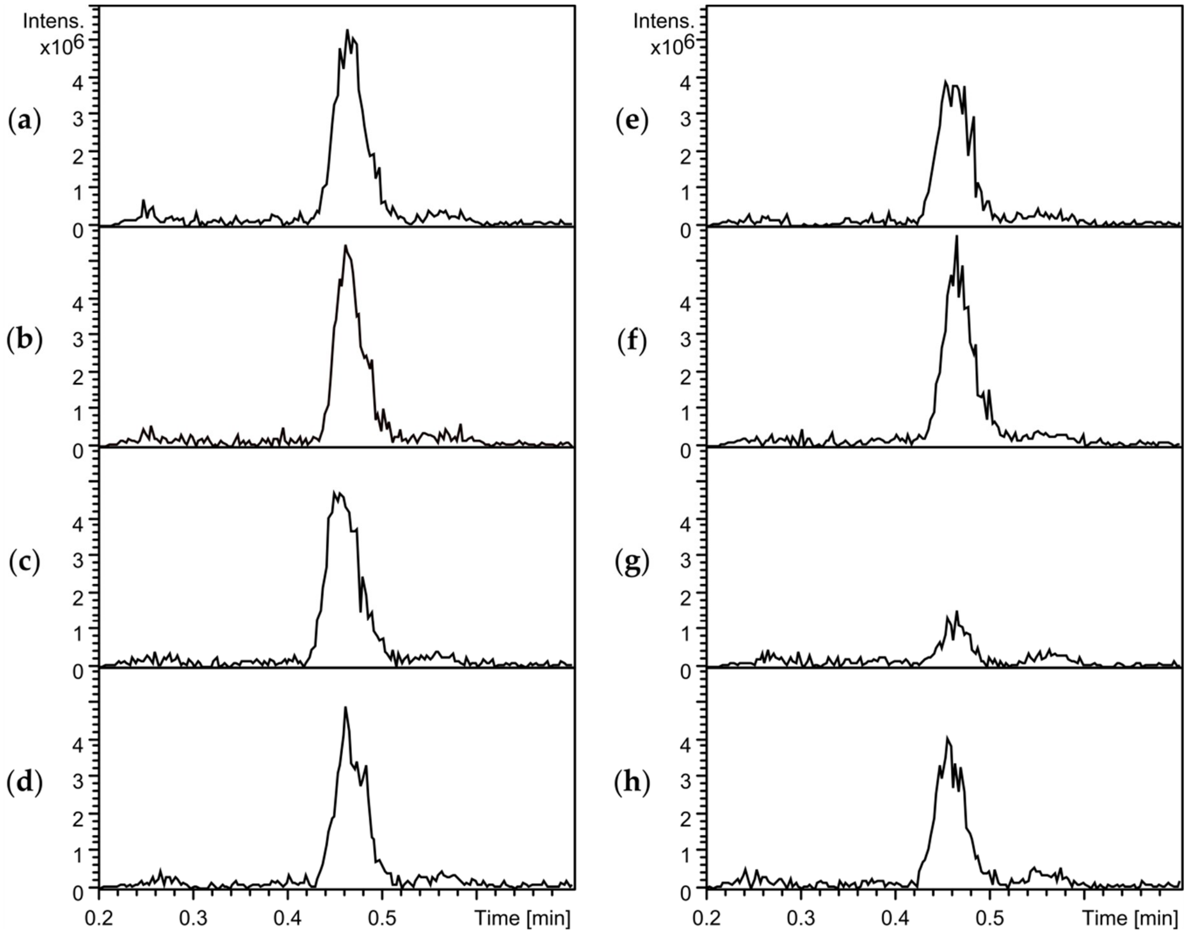

Figure S6. HPLC-MS analysis of albucidin production by *Streptomyces albus* strain harboring 1K1 BAC and its derivatives with gene deletions. Extracted chromatograms (222.00 ± 0. 5 Da) of crude extracts from *S. albus* 1K1 (**a**), *S. albus* 1K1_KO7 (**b**), *S. albus* 1K1_KO8 (**c**), *S. albus* 1K1_KO9 (**d**), *S. albus* 1K1_ KO10 (**e**), *S. albus* 1K1_ KO11 (**f**), *S. albus* 1K1_ KO12 (**g**), *S. albus* 1K1_ KO13(**h**).

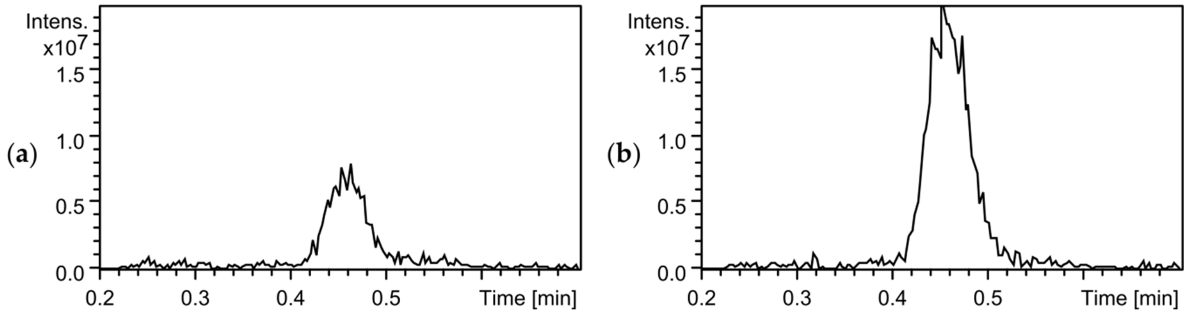

Figure S7. HPLC-MS analysis of albucidin production by *Streptomyces albus* strain harboring full length 1K1 BAC (**a**) and minimized 1K1_alb_act BAC, containing only transcriptionally activated *albA-C* operon (**b**). Extracted chromatograms (222.00 ± 0. 5 Da) are shown.

Table S1. Bacterial strains used in the study.

Strain	Description	Source
Streptomyces albus Del14	Cluster-free heterologous host strain	[19]
Streptomyces lividans TK24	Heterologous host strain	[20]
Escherichia coli GD10B	General coning host	[26]
Escherichia coli ET12567 pUB307	Donor strain for intergeneric conjugation	[27]

Table 2. Plasmids and BACs used in the study.

Plasmid/BAC	Description	Source
pUC19	Plasmid; General cloning vector; Used as a source of ampicillin resistance marker for RedET experiments	Thermo scientific
pACS-hyg	Plasmid; Contains promoter activation cassette; Used as a source of hygromycin resistance gene for RedET experiments	[16]
1K1	BAC; Contains albucidin cluster	[14]
2D4	BAC; Contains albucidin cluster	[14]
1K1_RS	Derivative of 1K1; contains substitution of the genes 17 – 28 (SACHL2_05523 - SACHL2_05512) with hygromycin resistance gene	This study
1K1_LS	Derivative of 1K1; contains substitution of the genes 1 – 13 (SACHL2_05539 - SACHL2_05527) with ampicillin resistance gene	This study
1K1_KO7	Derivative of 1K1; contains substitution of the gene 7 (SACHL2_05533) with ampicillin resistance gene	This study
1K1_KO8	Derivative of 1K1; contains substitution of the gene 8 (SACHL2_05532) with ampicillin resistance gene	This study
1K1_KO9	Derivative of 1K1; contains substitution of the gene 9 (SACHL2_05531) with ampicillin resistance gene	This study
1K1_KO10	Derivative of 1K1; contains substitution of the gene 10 (SACHL2_05530) with ampicillin resistance gene	This study
1K1_KO11	Derivative of 1K1; contains substitution of the gene 11 (SACHL2_05529) with ampicillin resistance gene	This study
1K1_KO12	Derivative of 1K1; contains substitution of the gene 12 (SACHL2_05528) with ampicillin resistance gene	This study
1K1_KO13	Derivative of 1K1; contains substitution of the gene 13 (SACHL2_05527) with ampicillin resistance gene	This study
1K1_KO14	Derivative of 1K1; contains substitution of the gene 14 (SACHL2_05526) with ampicillin resistance gene	This study
1K1_KO15	Derivative of 1K1; contains substitution of the gene 15 (SACHL2_05525) with ampicillin resistance gene	This study
1K1_KO16	Derivative of 1K1; contains substitution of the gene 16 (SACHL2_05524) with ampicillin resistance gene	This study
1K1_RS2	Derivative of 1K1; contains substitution of the genes 17 – 28 (SACHL2_05523 - SACHL2_05512) with ampicillin resistance gene	This study
1K1_alb_act	Derivative of 1K1_RS2; contains substitution of the genes 1 – 14 (SACHL2_05539 - SACHL2_05526) with hygromycin resistance gene	This study

[1] The locus tags refer to the genome sequence of *S. albus* subsp. *chlorinus* NRRL B-24108 available under GenBank accession number VJOK00000000.

Table 3. Primers used in this study.

Primer	Sequence
LS-F	TCACGTCGATATCGGCAGCGGGTGGATCTCCTGCGCGGGGTGCCCGGCGCCGTC

	AGGTGGCACTTTTCG
LS-R	GAGGCCGTGCGTCCGGGATCGAGGCGGACCAGGCCGGAGGCGTACAGCGGAAG CTTTTACCAATGCTTAATCAGTG
RS-F	ATGACAGGACTCGGAAGTAGTGGACCGACGTTCCGGCTCGATGAGGAGTTTCTA GAAATACTTGACATATCACTGT
RS-R	TCACCGGCCGGTCGTGTGAGCGAGGTTCCGAACAGCCAGCTCGCGCAGCATCAG GCGCCGGGGGCGGTGT
KO14-F	ATGAGCCCGTCAACCGCCGGGGTCGACGCCCGCCGCTACACATGGTGCTGGTTT AAACCGTCAGGTGGCACTTTTCG
KO14-R	TCAGGCCCCGGACGCCGGCCGTCGTCCCTCGACATCGGGCAGGTAGTCCCAAGC TTGTTTAAACTTACCAATGCTTAATCAGTG
KO15-F	ATGACGCTCTTATCCACCGAGCTTGACGCCACGGCGGCCCTGCGGCTCAAGTTTA AACCGTCAGGTGGCACTTTTCG
KO15-R	TCATGCGGGCAGGCGGGAGAACAACCCCAGGTAGGCTTCCGTGTCGATCGAAGC TTGTTTAAACTTACCAATGCTTAATCAGTG
KO16-F	ATGTCCCCGGTTGAGGCCGATGTGCGCACGTTGGAGGACAGGCTCAGCAGGTTT AAACCGTCAGGTGGCACTTTTCG
KO16-R	CTATTCGGCGACGCACTGCTCGGGCGGATTGATCAGCGTCCAGTCCGTTCAAGCT TGTTTAAACTTACCAATGCTTAATCAGTG
KO13-F	ATGCAAGGAGTGACTCTCGGCACCAAGTCCCTTGACCGAAAAAGCGTGGAGTTT AAACCGTCAGGTGGCACTTTTCG
KO13-R	TCATGAGGCCGTGCGTCCGGGATCGAGGCGGACCAGGCCGGAGGCGTACAAAG CTTGTTTAAACTTACCAATGCTTAATCAGTG
KO12-F	GCGGAAAACCGTCTCTTCCTGGATATCGACTGGCGCGAGGGCGGTTACCCAAGC TTGTTTAAACCGTCAGGTGGCACTTTTCG
KO12-R	TCAGAACAACGAGGGCTGGACGGCGCCGACCTCGGACGTGTAGTCACCCAGTTT AAACTTACCAATGCTTAATCAGTG
KO11-F	ATGGCCGAATTCAGCTTGTCACTCGACGGTTCTCTGGCGAATTCCCGCGAGTTTA AACCGTCAGGTGGCACTTTTCG
KO11-R	CCCGGTCTCGCTTTCGCGGTCCAGCCCCAGCAGATCGAGCAGCTCCTGGTAAGCT TGTTTAAACTTACCAATGCTTAATCAGTG
KO10-F	ATGTTGATCGCGTTCGAGGGAATCGACGGCGCCGGAAAGACCACGGCCGTAAG CTTGTTTAAACCGTCAGGTGGCACTTTTCG
KO10-R	TCATCGATCGGTCAGAAAGCCGTCCACCAGCGCCGCGACCTCGTCCGCCAGTTT AAACTTACCAATGCTTAATCAGTG
KO9-F	TTGGAGACACAGGCCAGCGACACGATCGAAGAATTCGACGTCGACCCCGAAAG CTTGTTTAAACCGTCAGGTGGCACTTTTCG
KO9-R	TCACCGGAGGGGGGCGTCACGGAACCGTTCCATGGTGACGCGTATGTGGTGTTT AAACTTACCAATGCTTAATCAGTG
KO8-F	GTGACGCCCCCCTCCGGTGAGAGCGGGACCAGGCACGCGGCCCTTGAGCGAAG CTTGTTTAAACCGTCAGGTGGCACTTTTCG

KO8-R	TCAGGTGCCAGCGCACAGGAACTCCTCCCATCGACGTACGTGAAGCGTCAGTTT AAACTTACCAATGCTTAATCAGTG
KO7-F	GTGCGCTGGCACCTGACCGGCATCACCGGTAAGTGCGTCCACCCCGGGTTAAGC TTGTTTAAACCGTCAGGTGGCACTTTTCG
KO7-R	CAGCTCGCTCCAGTGGGCGGAGAGGAGTTCGGAGGGGTCGGTCAGGTGGTGTTT AAACTTACCAATGCTTAATCAGTG
RS2-F	ATGACCGGTCAACCGGCGAGCGGGGAGCTCGACATGGTGCGCAGCGCCGTTCTA GACGTCAGGTGGCACTTTTCG
RS2-R	TCACCGGCCGGTCGTGTGAGCGAGGTTCCGAACAGCCAGCTCGCGCAGCAAAGC TTGTTTAAACTTACCAATGCTTAATCAGT
ACT-F	TCACGTCGATATCGGCAGCGGGTGGATCTCCTGCGCGGGGTGCCCGGCGCATTTA AATAATACTTGACATATCACTGT
ACT-R	TCGGTGGATAAGAGCGTCATACTCCGTCCTTCGTCTGCGGTTTGAGCCCATCAGG CGCCGGGGGCGGTGT
